# Supplementary material for: Fast and Economic Microarray-Based Detection of Species-, Resistance-, and Virulence-Associated Genes in Clinical Strains of Vancomycin-Resistant Enterococci (VRE)
Source: Sensors (Basel). 2024 Oct 8;24(19):6476. doi: 10.3390/s24196476 (PMC11479252; doi:10.3390/s24196476)
Supplement: Supplementary file 1 [file sensors-24-06476-s001.zip › Supplemental File S1.pdf]

Table S1: *A list of targets present in VRE-01 (the first generation) microarrays and their corresponding position marker as they appear on the layout and the microarray.*

**Number**	**Well**	**Gene Names**	**Probe Sequence (5´3´direction)**	**Target description**
1	A1	hp_0_aac6	CTGCCGAGCTGTCTCCATATTCTTCC	Aminoglycoside acetyltransferase (Aminoglycoside resistance)
2	B1	hp_2_aadA1	AGCGTTGCCTTGGTAGGTCC	Aminoglycoside resistance
3	C1	hp_0_aadK	CGTCGCTCAGGATTTTGCTAAAATGG	Aminoglycoside acetyltransferase (Aminoglycoside resistance)
4	D1	hp_2_ant6	AAGCGCAAGGGAGTATGATGATTGCT	Aminoglycoside nucleotidyltransferase (Aminoglycoside resistance)
5	E1	hp_0_aacAaphD	TGGTATGGATCAATTTATAGGAGAGCCA	Bifunctional enzyme Aac/Aph, aminoglycoside resistance
6	F1	hp_2_aphIIIa	ACAGCCGGTATAAAGGGACCACC	Aminoglycoside phosphotransferase (Aminoglycoside resistance)
7	G1	hp_0_cat	AGTGAGGGAAATTTGGGTTATTGGGA	Chloramphenicol O-acetyltransferase (chloramphenicol resistance)
8	H1	hp_2_catA	ACCTGAAAACACAGTTCCTATTTCGAT	Chloramphenicol O-acetyltransferase type A (chloramphenicol resistance)
9	I1	hp_0_cfr2	GTGAAGCTCTAGCCAACCGTC	Linezolid resistance
10	J1	hp_0_cfrB	ACATTAGATGAGCATATACGAGTAACCTCA	Linezolid resistance
11	K1	hp_0_ermA	AAAACCAATTCCGACAGGCTTTGAAG	Macrolide resistance
12	L1	hp_1_ermB	AAACTTACCCGCCATACCACAGATGT	Macrolide resistance
13	M1	hp_5_ermG	CCATCCAAAACCTAAAGTGGATAGCGC	Macrolide resistance
14	N1	hp_4_ermT	TCGTTCACTAGCACTATTTTTAATGACAGA	Macrolide resistance
15	O1	hp_0_fexA	AGGGTGGAATGCCTTGTTTTGGTT	Florfenicol resistance
16	P1	hp_0_fexB	TTGGGTCACTTTTGTTCTGGGTTTAA	Florfenicol resistance
17	A2	hp_0_lnu(B)	AGTCAGATGAACGAATAACAGCTTGT	Lincosamide resistance
18	B2	hp_0_lsa(A)	AACTTACGAAAACTTGGGATGGAGCG	Dalfopristin-quinopristin resistance intrinsic in faecalis
19	C2	hp_0_lsa(E)	ACATGGTGGATTTTAGTGCTGGTCAG	Dalfopristin-quinopristin resistance intrinsic in faecalis
20	D2	hp_0_mefA	ACGAAATGCGCAGGCTATAGTCAGT	Macrolide efflux
21	E2	hp_5_optrA	TGTGGAAAAACAACCTTGCTAAAAGC	Oxazolidinone
22	F2	hp_M_pbp5_1400_t-xxx-gtc	CTCGTGTTAGTGATGTGTCACAAGTAG	Penicillin binding protein 5
23	G2	hp_M_pbp5_1400_c-xxx-atc	CGCGTGTCAGTGATGTATCAC	Penicillin binding protein 5
24	H2	hp_M_pbp5_1400_c-tga-atc	GCGTGTCAGTGATGATGTATCAC	Penicillin binding protein 5

25	I2	hp_M_pbp5_1400_c-tag-atc	CGCGTGTCAGTAGTGATGTATCAC	Penicillin binding protein 5
26	J2	hp_M_pbp5_1462_gc	GCGGCACAAGAAACGTTG	Penicillin binding protein 5
27	K2	hp_0_poxtA	TCAGGCGGACAAAAATCCAAAATGGC	Linezolid resistance
28	L2	hp_0_qnr	AGTTCGTTAATAGAGGAAGTTGTTCTGG	Quinolone resistance
29	M2	hp_0_tet_K	CCTGGAACCATGAGTGTTATTGTTTTTGGT	Tetracycline resistance
30	N2	hp_0_tet_L	ACAAATATCAGGAATGACAGCACGCT	Tetracycline resistance
31	O2	hp_0_tet_M	GCGATTACAGAATTAGGAAGCGTGGA	Tetracycline resistance through ribosomal protection
32	P2	hp_0_tet_O	ATGACGATATGGAACAGTGGGATGCG	Tetracycline resistance through ribosomal protection
33	A3	hp_0_tet_S	AGAGCCATATCTTAGCTTCGAAATTTATGT	Tetracycline resistance through ribosomal protection
34	B3	hp_0_vanA	GGCAAGTCAGGTGAAGATGGATCC	D-alanine--D-lactate ligase
35	C3	hp_0_vanH_1	ACGATCCGAACATCTTTCAAAGATTTTC	D-alanine--D-serine ligase
36	D3	hp_0_vanH_2	CAGGATGAGGCAAATGCTTTCCGC	D-lactate dehydrogenase
37	E3	hp_0_vanH_3	GCTTCATGTGCCGCTCAATACG	D-lactate dehydrogenase
38	F3	hp_0_vanR	TACTATACCGCCAAAGAAGCATTGGA	*Van* two component sensor/regulator, transcriptional regulator
39	G3	hp_2_vanR-B	CGAAAACCAAATCCGGGCGTTTGAT	*Van* two component sensor/regulator, transcriptional regulator
40	H3	hp_0_vanR-G	TGCTGGAAGTATATTTAACGAGTGACGGT	*Van* two component sensor/regulator, transcriptional regulator
41	I3	hp_0_vanS-B	ATTCTGAATCAGTTCCACCAATGGCT	*Van* two component sensor/regulator, sensor histidine kinase
42	J3	hp_0_vanS-CE	GATCAACGAGCCGCCAAAGAAGC	*Van* two component sensor/regulator, sensor histidine kinase
43	K3	hp_4_vanS-FM	CGTACGCCGCTAACTTCTGTTTTAGG	*Van* two component sensor/regulator, sensor histidine kinase
44	L3	hp_3_vanS-G	CTTGAAGACGCCTTTGACTTCAGTGA	*Van* two component sensor/regulator, sensor histidine kinase
45	M3	hp_6_vanT-C1	ACAGCTAAAAGTCTATCTATTAGCCACAC	Membrane-bound serine racemase
46	N3	hp_0_vanT-C234	ATCAGCCGCCTAGGTTCCCG	Membrane-bound serine racemase
47	O3	hp_0_vanU	GGCGAGCTTCGTGAGGCTG	Transcriptional regulator
48	P3	hp_6_vanW	CATGTCGGGCGGCGGTATGTG	Vancomycin resistance protein W
49	A4	hp_9_vanX	GTGTTCGTTGGGATGCCAAGTACG	D-Ala-D-Ala dipeptidase
50	B4	hp_2_vanXY	ACCATGGCATTTTCGTTATGTCGGTC	Bifunctional D,D-carboxypeptidase/D,D-dipeptidase
51	C4	hp_7_vanY	AGTGCGATTATGAAAGAAAAGAATTTCGT	D-Ala-D-Ala carboxypeptidase
52	D4	hp_6_vanYB	CAAGTTACGGAGATCAGCCTGGAGC	D-Ala-D-Ala carboxypeptidase

53	E4	hp_0_vanYD	AAGCAGGTTTCAGGACAGCGTTGATA	Putative peptidase
54	F4	hp_0_vanZ	TCTCGTCCCTTTTTCTACTTTTTCTTTTGA	Transmembrane teicoplanin resistance protein
55	G4	hp_7_vatD	ATGCCAGGAGTAAAAATCGGGGATGG	Quinupristin-/Dalfopristin & streptogramin-resistance
56	H4	hp_5_vatE	CCACCGATTTTGAGAAACACGTTACCC	Quinupristin-/Dalfopristin & streptogramin-resistance
57	I4	hp_1_vgbA	ACATTGCCTAACCCAGATTCAGCACC	Quinupristin-/Dalfopristin & streptogramin-resistance
58	J4	hp_1_efmM	TAAAATCTTCGCACGTTTCGGAAAGA	Ribosomal RNA (rRNA) methyltransferase
59	K4	hp_7_ace1	ATGTGGAAATGCCAACAGAAGAAAGT	Collagen-binding microbial surface components recognizing adhesive matrix molecules (MSCRAMM) in faecalis
60	L4	hp_3_ace2	ATTGTTGCAACTGCCAATGATCGTGT	Collagen-binding microbial surface components recognizing adhesive matrix molecules (MSCRAMM) in faecalis
61	M4	hp_4_acm	TGTTGGATATCTTCCCAAGTCACTGA	Collagen-binding microbial surface components recognizing adhesive matrix molecules (MSCRAMM) in faecium
62	N4	hp_0_asp1	TTCAGCTTTTCAAGAACAATCGAGAAC	Factor associated with rabbit endocarditis and survival in PMNs
63	O4	hp_0_atlA	AGAACCTTTCCGTAAATATCCTTCTTACATG	Major autolysin
64	P4	hp_0_bepA_fruA	CAGAACAAGAAACACAAGCTGCAGAA	Permease
65	A5	hp_4_ccpA	ACTGTATTAAACAATTTACTTGCCAAGCAA	Collagen-binding MSCRAMM
66	B5	hp_4_ebpA	GAGATGGTGTCAGCCGATGAAAATGG	Endocarditis and biofilm-associated pili, Pilin subunit
67	C5	hp_0_ebpB	TGACGACGCAAAAGCATGGATTTACG	Endocarditis and biofilm-associated pili, Pilin subunit
68	D5	hp_2_ebpC	GGCACAACAGAAAACCTAGTTTCACC	Endocarditis and biofilm-associated pili, Pilin subunit
69	E5	hp_7_ecbA-v1	GCAAATTTAAAGAAATCAAAGCCAAAGCG	Collagen binding MSCRAMM
70	F5	hp_0_fss3	GGCAAGTTTGAAAATCTTCCTTATGGGG	Fss3 (surface protein)
71	G5	hp_6_eep	GCCGTTATGATTTAGAAGATGAGCTGA	Protease said to play a role in Endocarditis
72	H5	hp_0_efaA	TGCTAAAAGTACACGTTGAAATTGTCC	Adhesion protein, plays role in endocarditis
73	I5	hp_0_efbA_Fnm	GCAAAACAACAAGGCGGAGAACTGAT	Adhesion protein, plays role in endocarditis
74	J5	hp_3_esp	CGAGTTAGCGGGAACAGGTCACAAAG	Enterococcal surface protein
75	K5	hp_0_fss1	GGAAATGAAGTCGTTGGAGCAGAACT	Fss1 (*E. faecalis* surface protein)
76	L5	hp_4_fss2	GCTGTTGGAAACACAGGTTATGCACC	Fss2 (*E. faecalis* surface protein)
77	M5	hp_9_gelE	TGGAAAAGGAGGCAATTTCTGAGGCA	Gelatinase E
78	N5	hp_6_hyl	AGAATATGATAATCGTCATGGTTCTCGC	Beta-N-acetylglucosaminidase hyaluronidase
79	O5	hp_1_pilA	ACAAGGCAGATTATGGTGATGTTGAGT	Major pilin subunit
80	P5	hp_1_pilE	TGGCGCGAAGTTTTCTGTTTATGATGT	Secreted surface / cell wall-associated LPXTG-like protein
81	A6	hp_5_pilF	CTGAAGTGGGAAGCTTCCGCTGA	Minor pilin subunit

82	B6	hp_1_sagA_faecium	TTGCGTCAAGAATCTGCACAATTAGT	Adhesion protein
83	C6	hp_4_sagA_faecalis	CGCGATGTCCAAGTGAATGGTCAAAG	Adhesion protein
84	D6	hp_3_scm	TGCATTGAACGTACAGTCACCAATCA	Collagen-binding MSCRAMM
85	E6	hp_0_srtA1	TCCCAGGAATGGGACAAGTCAAAGAT	Biofilm and pilus-associated sortase
86	F6	hp_7_srtA2	CGTCACGCGACTAGTTGTACAAGGAG	Biofilm and pilus-associated sortase
87	G6	hp_0_srtA2C	AGATTCACTGAATGATTTTCTTGCCCA	LPXTG specific sortase A2C
88	H6	hp_6_srtC1	ACTGGTGATATTACAGGAGGAAACGGT	Pilus-related sortase
89	I6	hp_0_srtC2	AAAGAAGCAATCGAAATGGGCGTCAA	Pilus-related sortase
90	J6	hp_6_bee1	AAAGTGACGGCTGATGGAGAAGCA	Biofilm enhancer in enterococci
91	K6	hp_2_bee2	GCCGAACCCTTAGTAGTGGCTTTACC	Biofilm enhancer in enterococci
92	L6	hp_0_bee3	GCTGGTAGTCAATTGATTGCTACTTACA	Biofilm enhancer in enterococci
93	M6	hp_6_cylA	AAAGCTGCTGACTTTCCAGATGG	Cytolysin A
94	N6	hp_1_Hp1	ACCACTTCCCCTTGATATGCCTCATG	Toll/ Interleukin 1 Receptor
95	O6	hp_5_prgB	GCGGCAGGAGACGTTTACAA	LPXTG anchored aggregation substance
96	P6	hp_0_prpA	TCAGGACTAGCAAAGGTTGTATCCTT	Fibronectin/ fibrinogen-binding MSCRAMM
97	A7	hp_0_tirE1	TCAGTAATTAAAATAATTGGGCAAAAACCAGA	Toll/ Interleukin 1 Receptor
98	B7	hp_2_tirE2	GCAGATATTTTTATATCTCACTCTCATGCTGA	Toll/ Interleukin 1 Receptor
99	C7	hp_5_EFCL_ddl	CCGTACGACTTTACCTGGTGAAGTGG	D-alanine-D-alanine ligase encoding gene for *E. faecalis*
100	D7	hp_0_EGAL_vanC2_3	AATACGGGGAAGATGGCAGTATCCAA	Vancomycin resistance found in *E. gallinarum* and *E. casseliflavus* (*van* operon C)
101	E7	hp_1_EGAL_vanC1	CGAATGAAGCCGGTTCTTCAAAAGGG	Vancomycin resistance found in *E. gallinarum (van* operon C)
102	F7	tetM-11,3	GCTCGGTTTCTCTTGGATACTTAAATC	Tetracycline resistance through ribosomal protection
103	G7	18,2-vanA	ATTGTACTGAACGAAGTCAATACTCTGC	D-alanine--D-lactate ligase (Vancomycin resistance)
104	H7	19,3-vanB	AAAGAAAGTATATCGGGTGCTTGGAT	Vancomycin resistance (*van* operon B)
105	I7	vanB_11	GCAAAGAAAGTATATCGGGTGCTTG	Vancomycin resistance (*van* operon B)
106	J7	20,3-vanZ	CCATTTACTGCTACTGGGAATTTCAG	Transmembrane teicoplanin resistance protein (*van* operon Z)
107	K7	hp_mefA_611	TGATTGCATCTATTACGGTAGCAATTGT	Tetracycline resistance through efflux pump action
108	L7	hp_ermB_611	CGAAATTGGAACAGGTAAAGGGCA	Macrolide resistance
109	M7	hp_ermB_612	TCGTGTCACTTTAATTCACCAAGATATTCT	Macrolide resistance
110	N7	1x Spotting buffer		
111	O7	Biotin-Marker		

Table S2: *A list of targets present in VRE-02 (the second generation) microarrays and their corresponding position marker as they appear on the layout and the microarray.*

**Number**	**Well**	**Gene designation**	**Probe sequence (5´3´direction)**	**Target description**
1	A1	hp_0_aac6	CTGCCGAGCTGTCTCCATATTCTTCC	Aminoglycoside acetyltransferase (Aminoglycoside resistance)
2	B1	hp_2_aadA1	AGCGTTGCCTTGGTAGGTCC	Aminoglycoside resistance
3	C1	hp_0_aadK	CGTCGCTCAGGATTTTGCTAAAATGG	Aminoglycoside acetyltransferase (Aminoglycoside resistance)
4	D1	hp_2_ant6	AAGCGCAAGGGAGTATGATGATTGCT	aminoglycoside nucleotidyltransferase (Aminoglycoside resistance)
5	E1	hp_0_aacAaphD	TGGTATGGATCAATTTATAGGAGAGCCA	Bifunctional enzyme Aac/Aph, aminoglycoside resistance
6	F1	hp_2_aphIIIa	ACAGCCGGTATAAAGGGACCACC	Aminoglycoside phosphotransferase (Aminoglycoside resistance)
7	G1	hp_0_cat	AGTGAGGGAAATTTGGGTTATTGGGA	Chloramphenicol O-acetyltransferase (chloramphenicol resistance)
8	H1	hp_2_catA	ACCTGAAAACACAGTTCCTATTTCGAT	Chloramphenicol O-acetyltransferase type A (chloramphenicol resistance)
9	I1	hp_0_cfr2	GTGAAGCTCTAGCCAACCGTC	Linezolid resistance
10	J1	hp_0_cfrB	ACATTAGATGAGCATATACGAGTAACCTCA	Linezolid resistance
11	K1	hp_0_ermA	AAAACCAATTCCGACAGGCTTTGAAG	Macrolide resistance
12	L1	hp_1_ermB	AAACTTACCCGCCATACCACAGATGT	Macrolide resistance
13	M1	hp_5_ermG	CCATCCAAAACCTAAAGTGGATAGCGC	Macrolide resistance
14	N1	hp_4_ermT	TCGTTCACTAGCACTATTTTTAATGACAGA	Macrolide resistance
15	O1	hp_0_fexA	AGGGTGGAATGCCTTGTTTTGGTT	Florfenicol resistance
16	P1	hp_0_fexB	TTGGGTCACTTTTGTTCTGGGTTTAA	Florfenicol resistance
17	A2	hp_0_lnu(B)	AGTCAGATGAACGAATAACAGCTTGT	Lincosamide resistance
18	B2	hp_0_lsa(A)	AACTTACGAAAACTTGGGATGGAGCG	Dalfopristin-quinupristin resistance intrinsic in faecalis
19	C2	hp_0_lsa(E)	ACATGGTGGATTTTAGTGCTGGTCAG	Dalfopristin-quinupristin resistance intrinsic in faecalis
20	D2	hp_0_mefA	ACGAAATGCGCAGGCTATAGTCAGT	Macrolide efflux
21	E2	hp_5_optrA	TGTGGAAAAACAACCTTGCTAAAAGC	Oxazolidinone
22	F2	hp_M_pbp5_1400_t-xxx-gtc	CTCGTGTTAGTGATGTGTCACAAGTAG	Penicillin binding protein 5
23	G2	hp_M_pbp5_1400_c-xxx-atc	CGCGTGTCAGTGATGTATCAC	Penicillin binding protein 5
24	H2	hp_M_pbp5_1400_c-tga-atc	GCGTGTCAGTGATGATGTATCAC	Penicillin binding protein 5
25	I2	hp_M_pbp5_1400_c-tag-atc	CGCGTGTCAGTAGTGATGTATCAC	Penicillin binding protein 5
26	J2	hp_M_pbp5_1462_gc	GCGGCACAAGAAACGTTG	Penicillin binding protein 5
27	K2	hp_0_poxtA	TCAGGCGGACAAAAATCCAAAATGGC	Linezolid resistance

28	L2	hp_0_qnr	AGTTCGTTAATAGAGGAAGTTGTTCTGG	Quinolone resistance
29	M2	hp_0_tet_K	CCTGGAACCATGAGTGTTATTGTTTTTGGT	Tetracycline resistance
30	N2	hp_0_tet_L	ACAAATATCAGGAATGACAGCACGCT	Tetracycline resistance
31	O2	hp_0_tet_M	GCGATTACAGAATTAGGAAGCGTGGA	Tetracycline resistance through ribosomal protection
32	P2	hp_0_tet_O	ATGACGATATGGAACAGTGGGATGCG	Tetracycline resistance through ribosomal protection
33	A3	hp_0_tet_S	AGAGCCATATCTTAGCTTCGAAATTTATGT	Tetracycline resistance through ribosomal protection
34	B3	hp_0_vanA	GGCAAGTCAGGTGAAGATGGATCC	D-alanine--D-lactate ligase (Glycopeptide resistance gene)
35	C3	hp_0_vanH_1	ACGATCCGAACATCTTTCAAAGATTTTC	D-alanine--D-serine ligase (Glycopeptide resistance gene)
36	D3	hp_0_vanH_2	CAGGATGAGGCAAATGCTTTCCGC	D-lactate dehydrogenase (Glycopeptide resistance gene)
37	E3	hp_0_vanH_3	GCTTCATGTGCCGCTCAATACG	D-lactate dehydrogenase (Glycopeptide resistance gene)
38	F3	hp_0_vanR	TACTATACCGCCAAAGAAGCATTGGA	*Van* two component sensor/regulator, transcriptional regulator (Glycopeptide resistance gene)
39	G3	hp_2_vanR-B	CGAAAACCAAATCCGGGCGTTTGAT	*Van* two component sensor/regulator, transcriptional regulator (Glycopeptide resistance gene)
40	H3	hp_0_vanR-G	TGCTGGAAGTATATTTAACGAGTGACGGT	*Van* two component sensor/regulator, transcriptional regulator (Glycopeptide resistance gene)
41	I3	hp_0_vanS-B	ATTCTGAATCAGTTCCACCAATGGCT	*Van* two component sensor/regulator, sensor histidine kinase (Glycopeptide resistance gene)
42	J3	hp_0_vanS-CE	GATCAACGAGCCGCCAAAGAAGC	*Van* two component sensor/regulator, sensor histidine kinase (Glycopeptide resistance gene)
43	K3	hp_4_vanS-FM	CGTACGCCGCTAACTTCTGTTTTAGG	*Van* two component sensor/regulator, sensor histidine kinase (Glycopeptide resistance gene)
44	L3	hp_3_vanS-G	CTTGAAGACGCCTTTGACTTCAGTGA	*Van* two component sensor/regulator, sensor histidine kinase (Glycopeptide resistance gene)
45	M3	hp_6_vanT-C1	ACAGCTAAAAGTCTATCTATTAGCCACAC	Membrane-bound serine racemase (Glycopeptide resistance gene)
46	N3	hp_0_vanT-C234	ATCAGCCGCCTAGGTTCCCG	Membrane-bound serine racemase (Glycopeptide resistance gene)
47	O3	hp_0_vanU	GGCGAGCTTCGTGAGGCTG	Transcriptional regulator (Glycopeptide resistance gene)
48	P3	hp_6_vanW	CATGTCGGGCGGCGGTATGTG	Vancomycin resistance protein W (Glycopeptide resistance gene)

49	A4	hp_9_vanX	GTGTTCGTTGGGATGCCAAGTACG	D-Ala-D-Ala dipeptidase (Glycopeptide resistance gene)
50	B4	hp_2_vanXY	ACCATGGCATTTTCGTTATGTCGGTC	bifunctional D,D-carboxypeptidase/D,D-dipeptidase (Glycopeptide resistance gene)
51	C4	hp_7_vanY	AGTGCGATTATGAAAGAAAAGAATTTCGT	D-Ala-D-Ala carboxypeptidase (Glycopeptide resistance gene)
52	D4	hp_6_vanYB	CAAGTTACGGAGATCAGCCTGGAGC	D-Ala-D-Ala carboxypeptidase (Glycopeptide resistance gene)
53	E4	hp_0_vanYD	AAGCAGGTTTCAGGACAGCGTTGATA	Putative peptidase (Glycopeptide resistance gene)
54	F4	hp_0_vanZ	TCTCGTCCCTTTTTCTACTTTTTCTTTTGA	Transmembrane teicoplanin resistance protein (Glycopeptide resistance gene)
55	G4	hp_7_vatD	ATGCCAGGAGTAAAAATCGGGGATGG	Quinupristin-/Dalfopristin & streptogramin-resistance
56	H4	hp_5_vatE	CCACCGATTTTGAGAAACACGTTACCC	Quinupristin-/Dalfopristin & streptogramin-resistance
57	I4	hp_1_vgbA	ACATTGCCTAACCCAGATTCAGCACC	Quinupristin-/Dalfopristin & streptogramin-resistance
58	J4	hp_1_efmM	TAAAATCTTCGCACGTTTCGGAAAGA	Ribosomal RNA (rRNA) methyltransferase
59	K4	hp_7_ace1	ATGTGGAAATGCCAACAGAAGAAAGT	Collagen-binding microbial surface components recognizing adhesive matrix molecules (MSCRAMM) in faecalis
60	L4	hp_3_ace2	ATTGTTGCAACTGCCAATGATCGTGT	Collagen-binding microbial surface components recognizing adhesive matrix molecules (MSCRAMM) in faecalis
61	M4	hp_4_acm	TGTTGGATATCTTCCCAAGTCACTGA	Collagen-binding microbial surface components recognizing adhesive matrix molecules (MSCRAMM) in faecium
62	N4	hp_0_asp1	TTCAGCTTTTCAAGAACAATCGAGAAC	Factor associated with rabbit endocarditis and survival in PMNs
63	O4	hp_0_atlA	AGAACCTTTCCGTAAATATCCTTCTTACATG	Major autolysin
64	P4	hp_0_bepA_fruA	CAGAACAAGAAACACAAGCTGCAGAA	Permease
65	A5	hp_4_ccpA	ACTGTATTAAACAATTTACTTGCCAAGCAA	Collagen-binding MSCRAMM
66	B5	hp_4_ebpA	GAGATGGTGTCAGCCGATGAAAATGG	Endocarditis and biofilm-associated pili, Pilin subunit
67	C5	hp_0_ebpB	TGACGACGCAAAAGCATGGATTTACG	Endocarditis and biofilm-associated pili, Pilin subunit
68	D5	hp_2_ebpC	GGCACAACAGAAAACCTAGTTTCACC	Endocarditis and biofilm-associated pili, Pilin subunit
69	E5	hp_7_ecbA-v1	GCAAATTTAAAGAAATCAAAGCCAAAGCG	Collagen binding MSCRAMM
70	F5	hp_0_fss3	GGCAAGTTTGAAAATCTTCCTTATGGGG	Fss3 (surface protein)
71	G5	hp_6_eep	GCCGTTATGATTTAGAAGATGAGCTGA	Protease said to play a role in Endocarditis
72	H5	hp_0_efaA	TGCTAAAAGTACACGTTGAAATTGTCC	Adhesion protein, plays role in endocarditis

73	I5	hp_0_efbA_Fnm	GCAAAACAACAAGGCGGAGAACTGAT	Adhesion protein, plays role in endocarditis
74	J5	hp_3_esp	CGAGTTAGCGGGAACAGGTCACAAAG	Enterococcal surface protein
75	K5	hp_0_fss1	GGAAATGAAGTCGTTGGAGCAGAACT	Fss1 (*E. faecalis* surface protein)
76	L5	hp_4_fss2	GCTGTTGGAAACACAGGTTATGCACC	Fss2 (*E. faecalis* surface protein)
77	M5	hp_9_gelE	TGGAAAAGGAGGCAATTTCTGAGGCA	Gelatinase E
78	N5	hp_6_hyl	AGAATATGATAATCGTCATGGTTCTCGC	Beta-N-acetylglucosaminidase hyaluronidase
79	O5	hp_1_pilA	ACAAGGCAGATTATGGTGATGTTGAGT	Major pilin subunit
80	P5	hp_1_pilE	TGGCGCGAAGTTTTCTGTTTATGATGT	Secreted surface / cell wall-associated LPXTG-like protein
81	A6	hp_5_pilF	CTGAAGTGGGAAGCTTCCGCTGA	Minor pilin subunit
82	B6	hp_1_sagA_faecium	TTGCGTCAAGAATCTGCACAATTAGT	Adhesion protein
83	C6	hp_4_sagA_faecalis	CGCGATGTCCAAGTGAATGGTCAAAG	Adhesion protein
84	D6	hp_3_scm	TGCATTGAACGTACAGTCACCAATCA	Collagen-binding MSCRAMM
85	E6	hp_0_srtA1	TCCCAGGAATGGGACAAGTCAAAGAT	Biofilm and pilus-associated sortase
86	F6	hp_7_srtA2	CGTCACGCGACTAGTTGTACAAGGAG	Biofilm and pilus-associated sortase
87	G6	hp_0_srtA2C	AGATTCACTGAATGATTTTCTTGCCCA	LPXTG specific sortase A2C
88	H6	hp_6_srtC1	ACTGGTGATATTACAGGAGGAAACGGT	Pilus-related sortase
89	I6	hp_0_srtC2	AAAGAAGCAATCGAAATGGGCGTCAA	Pilus-related sortase
90	J6	hp_6_bee1	AAAGTGACGGCTGATGGAGAAGCA	Biofilm enhancer in enterococci
91	K6	hp_2_bee2	GCCGAACCCTTAGTAGTGGCTTTACC	Biofilm enhancer in enterococci
92	L6	hp_0_bee3	GCTGGTAGTCAATTGATTGCTACTTACA	Biofilm enhancer in enterococci
93	M6	hp_6_cylA	AAAGCTGCTGACTTTCCAGATGG	Cytolysin A
94	N6	hp_1_Hp1	ACCACTTCCCCTTGATATGCCTCATG	Toll/ Interleukin 1 Receptor
95	O6	hp_5_prgB	GCGGCAGGAGACGTTTACAA	LPXTG anchored aggregation substance
96	P6	hp_0_prpA	TCAGGACTAGCAAAGGTTGTATCCTT	Fibronectin/ fibrinogen-binding MSCRAMM
97	A7	hp_0_tirE1	TCAGTAATTAAAATAATTGGGCAAAAACCAGA	Toll/ Interleukin 1 Receptor
98	B7	hp_2_tirE2	GCAGATATTTTTATATCTCACTCTCATGCTGA	Toll/ Interleukin 1 Receptor
99	C7	hp_5_EFCL_ddl	CCGTACGACTTTACCTGGTGAAGTGG	D-alanine-D-alanine ligase encoding gene for *E. faecalis*
100	D7	hp_0_EGAL_vanC2_3	AATACGGGGAAGATGGCAGTATCCAA	Vancomycin resistance found in *E. gallinarum* and *E. casseliflavus* (van operon C)
101	E7	hp_1_EGAL_vanC1	CGAATGAAGCCGGTTCTTCAAAAGGG	Vancomycin resistance found in *E. gallinarum* (*van* operon C)

102	F7	hp_0_aad6	TCCCACCTGATTTAGATTATGGTTACAGT	Aminoglycoside resistance
103	G7	hp_1_vanC_cluster1	CCATTTTAAAATTATTGATGGAGCGAAAAGG	Glycopeptide resistance gene *(van* operon C)
104	H7	hp_0_vanC_cluster2	ACGGCAAAGAAGTTTTCCTGACTCCC	Glycopeptide resistance gene *(van* operon C)
105	I7	hp_0_vanC_cluster3	CAAGGATCGGTCGTTGCCTC	Glycopeptide resistance gene *(van* operon C)
106	J7	hp_0_ddlA_avium	AACACGATGTTGCGATTTTATCGGCT	D-alanine-D-alanine ligase encoding gene for *E. avium*
107	K7	hp_0_ddlA_casseliflavus	CATGAGGTATCACTGTTATCCGCTTT	D-alanine-D-alanine ligase encoding gene for *E. casseliflavus*
108	L7	hp_0_ddlA_durans	TTGACGAGTGCATGTGCTATGGACAA	D-alanine-D-alanine ligase encoding gene for *E. durans*
109	M7	hp_0_ddlA_faecalis	CCGTACGACTTTACCTGGTGAAGTGG	D-alanine-D-alanine ligase encoding gene for *E. faecalis*
110	N7	hp_0_ddlA_faecium	CAGGCGTATTGACCAGTGCATGTG	D-alanine-D-alanine ligase encoding gene for *E. faecium*
111	O7	hp_0_ddlA_hirae	GGACCTAATGGTGAAGATGGAACG	D-alanine-D-alanine ligase encoding gene for *E. hirae*
112	P7	hp_0_ddlA_raffinosus	GTCCAGAACACGATGTAGCGATTTTA	D-alanine-D-alanine ligase encoding gene for *E. raffinosus*
113	A8	hp_1_dnaA_avium	AACGATTAGTTTCTCGCTTTGCTTGG	Replicative DNA helicase in *E. avium*
114	B8	hp_0_dnaA_casseliflavus	TAGCTGCCAAAATTGTTGAAATGGGA	Replicative DNA helicase in *E. casseliflavus*
115	C8	hp_0_dnaA_durans	TCGCGGGCCAAATCGATTCG	Replicative DNA helicase in *E. durans*
116	D8	hp_1_dnaA_faecalis	ATATTACCACTAGTTTAGCGGCGGAC	Replicative DNA helicase in *E.faecalis*
117	E8	hp_0_dnaA_faecium	GGTAAAGGAAACCAGATGGCTCATGC	Replicative DNA helicase in *E. faecium*
118	F8	hp_3_dnaA_gallinarum	ACTCACTTGATGCATGCTATCGGACA	Replicative DNA helicase in *E. gallinarium*
119	G8	hp_0_dnaA_hirae	TCGTCGAAGTACCAAGTAAAATTCATAAAGA	Replicative DNA helicase in *E. hirae*
120	H8	hp_0_dnaA_raffinosus	CGTGTGAAAGCAATTGTAGTTCCCCG	Replicative DNA helicase in *E. raffinosus*
121	I8	hp_3_msrC_faecium	GAACAAGGGATGCTTCTAGACGAGCC	Macrolide efflux in *E. faecium*
122	J8	hp_0_recG_faecium	TGAAGTTGGCTTGTTACATGGTAAAATG	Super family 2 helicase recG in *E. faecium*
123	K8	hp_0_recG_faecalis	ACTTATTGACGTATTATCCCTTCCGC	Super family 2 helicase recG in *E. faecalis*
124	L8	hp_0_rpmB_faecalis	CGTTCACATGCGATGAACTCAACAAA	Ribosomal protein in *E. faecalis*
125	M8	hp_0_rpmB_faecium	AAAAAGTTTGGGTGTCAACTCGTGC	Ribosomal protein in *E. faecium*
126	N8	hp_1_tuf_faecalis	AGAATTAGTAGAAATGGAAGTTCGTGACTT	Encodes elongation factor in *E. faecalis*
127	O8	hp_0_tuf_faecium	GATGCACCAGGACACGCGGACTAT	Encodes elongation factor in *E. faecium*
128	P8	hp_2_sodA_faecalis	TAGGTCTTGACGTTTGGGAACATGCG	Superoxide dismutase in *E. faecalis*

129	A9	hp_0_sodA_faecium	TAGGTTTGGACGTTTGGGAACATGCT	Superoxide dismutase in *E. faecium*
130	B9	tetM-11,3	GCTCGGTTTCTCTTGGATACTTAAATC	Tetracycline resistance through ribosomal protection
131	C9	18,2-vanA	ATTGTACTGAACGAAGTCAATACTCTGC	D-alanine--D-lactate ligase (Glycopeptide resistance)
132	D9	19,3-vanB	AAAGAAAGTATATCGGGTGCTTGGAT	Vancomycin resistance (*van* operon B)
133	E9	vanB_11	GCAAAGAAAGTATATCGGGTGCTTG	Vancomycin resistance (*van* operon B)
134	F9	20,3-vanZ	CCATTTACTGCTACTGGGAATTTCAG	Transmembrane teicoplanin resistance protein (*van* operon Z)
135	G9	hp_mefA_611	TGATTGCATCTATTACGGTAGCAATTGT	Tetracycline resistance through efflux pump action
136	H9	hp_ermB_611	CGAAATTGGAACAGGTAAAGGGCA	Macrolide resistance
137	I9	hp_ermB_612	TCGTGTCACTTTAATTCACCAAGATATTCT	Macrolide resistance
138	J9	1x Spotting buffer		
139	K9	Biotin-Marker		

Figure S1: *The layout of the VRE-01 microarray had 19 × 19 grids, 361 spots, 0.2 mm spot spacing, maximum size: 3.68 × 3.68 mm.*

111	87	88	89	90	91	92	93	94	95	96	97	98	99	100	101	102	103	111
	70	71	72	73	74	75	76	77	78	79	80	81	82	83	84	85	86	
111	53	54	55	56	57	58	59	60	61	62	63	64	65	66	67	68	69	111
111	36	37	38	39	40	41	42	43	44	45	46	47	48	49	50	51	52	111
	19	20	21	22	23	24	25	26	27	28	29	30	31	32	33	34	35	111
	2	3	4	5	6	7	8	9	10	11	12	13	14	15	16	17	18	111
	95	96	97	98	99	100	101	102	103	104	105	106	107	108	109	110	1	111
	78	79	80	81	82	83	84	85	86	87	88	89	90	91	92	93	94	111
	61	62	63	64	65	66	67	68	69	70	71	72	73	74	75	76	77	
	44	45	46	47	48	49	50	51	52	53	54	55	56	57	58	59	60	
	27	28	29	30	31	32	33	34	35	36	37	38	39	40	41	42	43	
	10	11	12	13	14	15	16	17	18	19	20	21	22	23	24	25	26	
	103	104	105	106	107	108	109	110	1	2	3	4	5	6	7	8	9	
	86	87	88	89	90	91	92	93	94	95	96	97	98	99	100	101	102	
	69	70	71	72	73	74	75	76	77	78	79	80	81	82	83	84	85	
	52	53	54	55	56	57	58	59	60	61	62	63	64	65	66	67	68	
	35	36	37	38	39	40	41	42	43	44	45	46	47	48	49	50	51	
	18	19	20	21	22	23	24	25	26	27	28	29	30	31	32	33	34	
111	1	2	3	4	5	6	7	8	9	10	11	12	13	14	15	16	17	111

Figure S2: *The layout of the VRE-02 microarray 22 × 22 grids, 484 spots, 0.17 mm spot spacing, maximum size: 3.68 × 3.68 mm.*

139																					139
	121	122	123	124	125	126	127	128	129	130	131	132	133	134	135	136	137	138			
139	101	102	103	104	105	106	107	108	109	110	111	112	113	114	115	116	117	118	119	120	139
139	81	82	83	84	85	86	87	88	89	90	91	92	93	94	95	96	97	98	99	100	139
	61	62	63	64	65	66	67	68	69	70	71	72	73	74	75	76	77	78	79	80	139
	41	42	43	44	45	46	47	48	49	50	51	52	53	54	55	56	57	58	59	60	139
	21	22	23	24	25	26	27	28	29	30	31	32	33	34	35	36	37	38	39	40	139
	1	2	3	4	5	6	7	8	9	10	11	12	13	14	15	16	17	18	19	20	139
	121	122	123	124	125	126	127	128	129	130	131	132	133	134	135	136	137	138			
	101	102	103	104	105	106	107	108	109	110	111	112	113	114	115	116	117	118	119	120	
	81	82	83	84	85	86	87	88	89	90	91	92	93	94	95	96	97	98	99	100	
	61	62	63	64	65	66	67	68	69	70	71	72	73	74	75	76	77	78	79	80	
	41	42	43	44	45	46	47	48	49	50	51	52	53	54	55	56	57	58	59	60	
	21	22	23	24	25	26	27	28	29	30	31	32	33	34	35	36	37	38	39	40	
	1	2	3	4	5	6	7	8	9	10	11	12	13	14	15	16	17	18	19	20	
	121	122	123	124	125	126	127	128	129	130	131	132	133	134	135	136	137	138			
	101	102	103	104	105	106	107	108	109	110	111	112	113	114	115	116	117	118	119	120	
	81	82	83	84	85	86	87	88	89	90	91	92	93	94	95	96	97	98	99	100	
	61	62	63	64	65	66	67	68	69	70	71	72	73	74	75	76	77	78	79	80	
	41	42	43	44	45	46	47	48	49	50	51	52	53	54	55	56	57	58	59	60	
	21	22	23	24	25	26	27	28	29	30	31	32	33	34	35	36	37	38	39	40	
139	1	2	3	4	5	6	7	8	9	10	11	12	13	14	15	16	17	18	19	20	139

Table S3: *A list of target genes present on VRE-01 and VRE-02 microarrays including their melting temperatures, probe sequences, the length of the probe sequences, GC contents, and reverse complement of the probe sequences.*

The maximum, minimum, delta and average values of the lengths, GC contents and melting temperatures are included at the bottom of the list.

Number	Name	Probe sequence (5´3´direction)	Length	GC%	Tm_santalucia	ReverseComplement
1	hp_0_aac6	CTGCCGAGCTGTCTCCATATTCTTCC	26	53,85	62,25	GGAAGAATATGGAGACAGCTCGGCAG
2	hp_2_aadA1	AGCGTTGCCTTGGTAGGTCC	20	60,00	59,75	GGACCTACCAAGGCAACGCT
3		CGTCGCTCAGGATTTTGCTAAAATGG	26	46,15	60,55	CCATTTTAGCAAAATCCTGAGCGACG
4	hp_2_ant6	AAGCGCAAGGGAGTATGATGATTGCT	26	46,15	62,18	AGCAATCATCATACTCCCTTGCGCTT
5	hp_0_aacAaphD	TGGTATGGATCAATTTATAGGAGAGCCA	28	39,29	58,75	TGGCTCTCCTATAAATTGATCCATACCA
6	hp_2_aphIIIa	ACAGCCGGTATAAAGGGACCACC	23	56,52	61,38	GGTGGTCCCTTTATACCGGCTGT
7	hp_0_cat	AGTGAGGGAAATTTGGGTTATTGGGA	26	42,31	59,57	TCCCAATAACCCAAATTTCCCTCACT
8	hp_2_catA	ACCTGAAAACACAGTTCCTATTTCGAT	27	37,04	58,38	ATCGAAATAGGAACTGTGTTTTCAGGT
9	hp_0_cfr2	GTGAAGCTCTAGCCAACCGTC	21	57,14	58,34	GACGGTTGGCTAGAGCTTCAC
10	hp_0_cfrB	ACATTAGATGAGCATATACGAGTAACCTCA	30	36,67	58,95	TGAGGTTACTCGTATATGCTCATCTAATGT

11	hp_0_ermA	AAAACCAATTCCGACAGGCTTTGAAG	26	42,31	60,07	CTTCAAAGCCTGTCGGAATTGGTTTT
12	hp_1_ermB	AAACTTACCCGCCATACCACAGATGT	26	46,15	61,58	ACATCTGTGGTATGGCGGGTAAGTTT
13	hp_5_ermG	CCATCCAAAACCTAAAGTGGATAGCGC	27	48,15	61,57	GCGCTATCCACTTTAGGTTTTGGATGG
14	hp_4_ermT	TCGTTCACTAGCACTATTTTTAATGACAGA	30	33,33	58,58	TCTGTCATTAAAAATAGTGCTAGTGAACGA
15	hp_0_fexA	AGGGTGGAATGCCTTGTTTTGGTT	24	45,83	60,70	AACCAAAACAAGGCATTCCACCCT
16	hp_0_fexB	TTGGGTCACTTTTGTTCTGGGTTTAA	26	38,46	58,68	TTAAACCCAGAACAAAAGTGACCCAA
17	hp_0_lnu(B)	AGTCAGATGAACGAATAACAGCTTGT	26	38,46	58,26	ACAAGCTGTTATTCGTTCATCTGACT
18	hp_0_lsa(A)	AACTTACGAAAACTTGGGATGGAGCG	26	46,15	61,11	CGCTCCATCCCAAGTTTTCGTAAGTT
19	hp_0_lsa(E)	ACATGGTGGATTTTAGTGCTGGTCAG	26	46,15	60,63	CTGACCAGCACTAAAATCCACCATGT
20	hp_0_mefA	ACGAAATGCGCAGGCTATAGTCAGT	25	48,00	61,87	ACTGACTATAGCCTGCGCATTTCGT
21	hp_5_optrA	TGTGGAAAAACAACCTTGCTAAAAGC	26	38,46	58,82	GCTTTTAGCAAGGTTGTTTTTCCACA
22	hp_M_pbp5_1400_t-xxx-gtc	CTCGTGTTAGTGATGTGTCACAAGTAG	27	44,44	59,00	CTACTTGTGACACATCACTAACACGAG
23	hp_M_pbp5_1400_c-xxx-atc	CGCGTGTCAGTGATGTATCAC	21	52,38	56,81	GTGATACATCACTGACACGCG
24	hp_M_pbp5_1400_c-tga-atc	GCGTGTCAGTGATGATGTATCAC	23	47,83	57,10	GTGATACATCATCACTGACACGC
25	hp_M_pbp5_1400_c-tag-atc	CGCGTGTCAGTAGTGATGTATCAC	24	50,00	58,87	GTGATACATCACTACTGACACGCG
26	hp_M_pbp5_1462_gc	GCGGCACAAGAAACGTTG	18	55,56	55,56	CAACGTTTCTTGTGCCGC
27	hp_0_poxtA	TCAGGCGGACAAAAATCCAAAATGGC	26	46,15	62,27	GCCATTTTGGATTTTTGTCCGCCTGA
28	hp_0_qnr	AGTTCGTTAATAGAGGAAGTTGTTCTGG	28	39,29	58,43	CCAGAACAACTTCCTCTATTAACGAACT
29	hp_0_tet_K	CCTGGAACCATGAGTGTTATTGTTTTTGGT	30	40,00	61,56	ACCAAAAACAATAACACTCATGGTTCCAGG
30	hp_0_tet_L	ACAAATATCAGGAATGACAGCACGCT	26	42,31	60,46	AGCGTGCTGTCATTCCTGATATTTGT
31	hp_0_tet_M	GCGATTACAGAATTAGGAAGCGTGGA	26	46,15	60,56	TCCACGCTTCCTAATTCTGTAATCGC
32	hp_0_tet_O	ATGACGATATGGAACAGTGGGATGCG	26	50,00	62,26	CGCATCCCACTGTTCCATATCGTCAT
33	hp_0_tet_S	AGAGCCATATCTTAGCTTCGAAATTTATGT	30	33,33	58,51	ACATAAATTTCGAAGCTAAGATATGGCTCT
34	hp_0_vanA	GGCAAGTCAGGTGAAGATGGATCC	24	54,17	60,50	GGATCCATCTTCACCTGACTTGCC
35	hp_0_vanH_1	ACGATCCGAACATCTTTCAAAGATTTTC	28	35,71	58,29	GAAAATCTTTGAAAGATGTTCGGATCGT
36	hp_0_vanH_2	CAGGATGAGGCAAATGCTTTCCGC	24	54,17	62,30	GCGGAAAGCATTTGCCTCATCCTG
37	hp_0_vanH_3	GCTTCATGTGCCGCTCAATACG	22	54,55	60,09	CGTATTGAGCGGCACATGAAGC
38	hp_0_vanR	TACTATACCGCCAAAGAAGCATTGGA	26	42,31	59,52	TCCAATGCTTCTTTGGCGGTATAGTA

39	hp_2_vanR-B	CGAAAACCAAATCCGGGCGTTTGAT	25	48,00	62,24	ATCAAACGCCCGGATTTGGTTTTCG
40	hp_0_vanR-G	TGCTGGAAGTATATTTAACGAGTGACGGT	29	41,38	61,43	ACCGTCACTCGTTAAATATACTTCCAGCA
41	hp_0_vanS-B	ATTCTGAATCAGTTCCACCAATGGCT	26	42,31	60,13	AGCCATTGGTGGAACTGATTCAGAAT
42	hp_0_vanS-CE	GATCAACGAGCCGCCAAAGAAGC	23	56,52	62,21	GCTTCTTTGGCGGCTCGTTGATC
43	hp_4_vanS-FM	CGTACGCCGCTAACTTCTGTTTTAGG	26	50,00	61,45	CCTAAAACAGAAGTTAGCGGCGTACG
44	hp_3_vanS-G	CTTGAAGACGCCTTTGACTTCAGTGA	26	46,15	60,78	TCACTGAAGTCAAAGGCGTCTTCAAG
45	hp_6_vanT-C1	ACAGCTAAAAGTCTATCTATTAGCCACAC	29	37,93	58,30	GTGTGGCTAATAGATAGACTTTTAGCTGT
46	hp_0_vanT-C234	ATCAGCCGCCTAGGTTCCCG	20	65,00	61,45	CGGGAACCTAGGCGGCTGAT
47	hp_0_vanU	GGCGAGCTTCGTGAGGCTG	19	68,42	61,00	CAGCCTCACGAAGCTCGCC
48	hp_6_vanW	CATGTCGGGCGGCGGTATGTG	21	66,67	63,58	CACATACCGCCGCCCGACATG
49	hp_9_vanX	GTGTTCGTTGGGATGCCAAGTACG	24	54,17	61,70	CGTACTTGGCATCCCAACGAACAC
50	hp_2_vanXY	ACCATGGCATTTTCGTTATGTCGGTC	26	46,15	61,43	GACCGACATAACGAAAATGCCATGGT
51	hp_7_vanY	AGTGCGATTATGAAAGAAAAGAATTTCGT	29	31,03	58,22	ACGAAATTCTTTTCTTTCATAATCGCACT
52	hp_6_vanYB	CAAGTTACGGAGATCAGCCTGGAGC	25	56,00	62,36	GCTCCAGGCTGATCTCCGTAACTTG
53	hp_0_vanYD	AAGCAGGTTTCAGGACAGCGTTGATA	26	46,15	62,01	TATCAACGCTGTCCTGAAACCTGCTT
54	hp_0_vanZ	TCTCGTCCCTTTTTCTACTTTTTCTTTTGA	30	33,33	59,00	TCAAAAGAAAAAGTAGAAAAAGGGACGAGA
55	hp_7_vatD	ATGCCAGGAGTAAAAATCGGGGATGG	26	50,00	62,20	CCATCCCCGATTTTTACTCCTGGCAT
56	hp_5_vatE	CCACCGATTTTGAGAAACACGTTACCC	27	48,15	61,86	GGGTAACGTGTTTCTCAAAATCGGTGG
57	hp_1_vgbA	ACATTGCCTAACCCAGATTCAGCACC	26	50,00	62,57	GGTGCTGAATCTGGGTTAGGCAATGT
58	hp_1_efmM	TAAAATCTTCGCACGTTTCGGAAAGA	26	38,46	59,21	TCTTTCCGAAACGTGCGAAGATTTTA
59	hp_7_ace1	ATGTGGAAATGCCAACAGAAGAAAGT	26	38,46	59,07	ACTTTCTTCTGTTGGCATTTCCACAT
60	hp_3_ace2	ATTGTTGCAACTGCCAATGATCGTGT	26	42,31	61,77	ACACGATCATTGGCAGTTGCAACAAT
61	hp_4_acm	TGTTGGATATCTTCCCAAGTCACTGA	26	42,31	58,90	TCAGTGACTTGGGAAGATATCCAACA
62	hp_0_asp1	TTCAGCTTTTCAAGAACAATCGAGAAC	27	37,04	58,21	GTTCTCGATTGTTCTTGAAAAGCTGAA
63	hp_0_atlA	AGAACCTTTCCGTAAATATCCTTCTTACATG	31	35,48	58,82	CATGTAAGAAGGATATTTACGGAAAGGTTCT
64	hp_0_bepA_fruA	CAGAACAAGAAACACAAGCTGCAGAA	26	42,31	60,02	TTCTGCAGCTTGTGTTTCTTGTTCTG
65	hp_4_ccpA	ACTGTATTAAACAATTTACTTGCCAAGCAA	30	30,00	58,47	TTGCTTGGCAAGTAAATTGTTTAATACAGT
66	hp_4_ebpA	GAGATGGTGTCAGCCGATGAAAATGG	26	50,00	61,49	CCATTTTCATCGGCTGACACCATCTC
67	hp_0_ebpB	TGACGACGCAAAAGCATGGATTTACG	26	46,15	62,10	CGTAAATCCATGCTTTTGCGTCGTCA

68	hp_2_ebpC	GGCACAACAGAAAACCTAGTTTCACC	26	46,15	60,07	GGTGAAACTAGGTTTTCTGTTGTGCC
69	hp_7_ecbA-v1	GCAAATTTAAAGAAATCAAAGCCAAAGCG	29	34,48	59,31	CGCTTTGGCTTTGATTTCTTTAAATTTGC
70	hp_0_fss3	GGCAAGTTTGAAAATCTTCCTTATGGGG	28	42,86	60,22	CCCCATAAGGAAGATTTTCAAACTTGCC
71	hp_6_eep	GCCGTTATGATTTAGAAGATGAGCTGA	27	40,74	58,67	TCAGCTCATCTTCTAAATCATAACGGC
72	hp_0_efaA	TGCTAAAAGTACACGTTGAAATTGTCC	27	37,04	58,21	GGACAATTTCAACGTGTACTTTTAGCA
73	hp_0_efbA_Fnm	GCAAAACAACAAGGCGGAGAACTGAT	26	46,15	61,98	ATCAGTTCTCCGCCTTGTTGTTTTGC
74	hp_3_esp	CGAGTTAGCGGGAACAGGTCACAAAG	26	53,85	62,98	CTTTGTGACCTGTTCCCGCTAACTCG
75	hp_0_fss1	GGAAATGAAGTCGTTGGAGCAGAACT	26	46,15	60,84	AGTTCTGCTCCAACGACTTCATTTCC
76	hp_4_fss2	GCTGTTGGAAACACAGGTTATGCACC	26	50,00	62,24	GGTGCATAACCTGTGTTTCCAACAGC
77	hp_9_gelE	TGGAAAAGGAGGCAATTTCTGAGGCA	26	46,15	62,21	TGCCTCAGAAATTGCCTCCTTTTCCA
78	hp_6_hyl	AGAATATGATAATCGTCATGGTTCTCGC	28	39,29	58,75	GCGAGAACCATGACGATTATCATATTCT
79	hp_1_pilA	ACAAGGCAGATTATGGTGATGTTGAGT	27	40,74	60,05	ACTCAACATCACCATAATCTGCCTTGT
80	hp_1_pilE	TGGCGCGAAGTTTTCTGTTTATGATGT	27	40,74	61,41	ACATCATAAACAGAAAACTTCGCGCCA
81	hp_5_pilF	CTGAAGTGGGAAGCTTCCGCTGA	23	56,52	62,09	TCAGCGGAAGCTTCCCACTTCAG
82	hp_1_sagA_faecium	TTGCGTCAAGAATCTGCACAATTAGT	26	38,46	59,20	ACTAATTGTGCAGATTCTTGACGCAA
83	hp_4_sagA_faecalis	CGCGATGTCCAAGTGAATGGTCAAAG	26	50,00	62,11	CTTTGACCATTCACTTGGACATCGCG
84	hp_3_scm	TGCATTGAACGTACAGTCACCAATCA	26	42,31	60,57	TGATTGGTGACTGTACGTTCAATGCA
85	hp_0_srtA1	TCCCAGGAATGGGACAAGTCAAAGAT	26	46,15	61,04	ATCTTTGACTTGTCCCATTCCTGGGA
86	hp_7_srtA2	CGTCACGCGACTAGTTGTACAAGGAG	26	53,85	62,56	CTCCTTGTACAACTAGTCGCGTGACG
87	hp_0_srtA2C	AGATTCACTGAATGATTTTCTTGCCCA	27	37,04	58,86	TGGGCAAGAAAATCATTCAGTGAATCT
88	hp_6_srtC1	ACTGGTGATATTACAGGAGGAAACGGT	27	44,44	60,75	ACCGTTTCCTCCTGTAATATCACCAGT
89	hp_0_srtC2	AAAGAAGCAATCGAAATGGGCGTCAA	26	42,31	61,55	TTGACGCCCATTTCGATTGCTTCTTT
90	hp_6_bee1	AAAGTGACGGCTGATGGAGAAGCA	24	50,00	61,87	TGCTTCTCCATCAGCCGTCACTTT
91	hp_2_bee2	GCCGAACCCTTAGTAGTGGCTTTACC	26	53,85	62,37	GGTAAAGCCACTACTAAGGGTTCGGC
92	hp_0_bee3	GCTGGTAGTCAATTGATTGCTACTTACA	28	39,29	58,94	TGTAAGTAGCAATCAATTGACTACCAGC
93	hp_6_cylA	AAAGCTGCTGACTTTCCAGATGG	23	47,83	58,63	CCATCTGGAAAGTCAGCAGCTTT
94	hp_1_Hp1	ACCACTTCCCCTTGATATGCCTCATG	26	50,00	61,70	CATGAGGCATATCAAGGGGAAGTGGT
95	hp_5_prgB	GCGGCAGGAGACGTTTACAA	20	55,00	58,30	TTGTAAACGTCTCCTGCCGC

96	hp_0_prpA	TCAGGACTAGCAAAGGTTGTATCCTT	26	42,31	58,90	AAGGATACAACCTTTGCTAGTCCTGA
97	hp_0_tirE1	TCAGTAATTAAAATAATTGGGCAAAAACCAGA	32	28,12	58,41	TCTGGTTTTTGCCCAATTATTTTAATTACTGA
98	hp_2_tirE2	GCAGATATTTTTATATCTCACTCTCATGCTGA	32	34,38	58,93	TCAGCATGAGAGTGAGATATAAAAATATCTGC
99	hp_5_EFCL_ddl	CCGTACGACTTTACCTGGTGAAGTGG	26	53,85	62,29	CCACTTCACCAGGTAAAGTCGTACGG
100	hp_0_EGAL_vanC2_3	AATACGGGGAAGATGGCAGTATCCAA	26	46,15	60,98	TTGGATACTGCCATCTTCCCCGTATT
101	hp_1_EGAL_vanC1	CGAATGAAGCCGGTTCTTCAAAAGGG	26	50,00	62,07	CCCTTTTGAAGAACCGGCTTCATTCG
102	tetM-11-3	GCTCGGTTTCTCTTGGATACTTAAATC	27	40,74	57,52	GATTTAAGTATCCAAGAGAAACCGAGC
103	18-2-vanA	ATTGTACTGAACGAAGTCAATACTCTGC	28	39,29	58,95	GCAGAGTATTGACTTCGTTCAGTACAAT
104	19-3-vanB	AAAGAAAGTATATCGGGTGCTTGGAT	26	38,46	57,84	ATCCAAGCACCCGATATACTTTCTTT
105	vanB_11	GCAAAGAAAGTATATCGGGTGCTTG	25	44,00	58,02	CAAGCACCCGATATACTTTCTTTGC
106	20-3-vanZ	CCATTTACTGCTACTGGGAATTTCAG	26	42,31	57,43	CTGAAATTCCCAGTAGCAGTAAATGG
107	hp_mefA_611	TGATTGCATCTATTACGGTAGCAATTGT	28	35,71	58,78	ACAATTGCTACCGTAATAGATGCAATCA
108	hp_ermB_611	CGAAATTGGAACAGGTAAAGGGCA	24	45,83	59,07	TGCCCTTTACCTGTTCCAATTTCG
109	hp_ermB_612	TCGTGTCACTTTAATTCACCAAGATATTCT	30	33,33	58,57	AGAATATCTTGGTGAATTAAAGT[illegible]
			Length	GC%	Tm_santalucia	
Minimum			18	28,12	55,56	
Maximum			32	68,42	63,58	
Delta (Maximum-Minimum)			14	40,30	8,03	
Average			26	45,14	60,20	

***Tm – melting temperature, GC%- GC content**
